# Digitale Interventionen bei Psychosen: aktuelle Chancen und Herausforderungen

**DOI:** 10.1007/s00115-025-01883-x

**Published:** 2025-08-11

**Authors:** Melina Luker, Mar Rus-Calafell

**Affiliations:** 1 Forschungs- und Behandlungszentrum für psychische Gesundheit, Fakultät für Psychologie, Ruhr-Universität Bochum, Bochum, Deutschland; 2 Partnerstandort Bochum/Marburg, Deutsches Zentrum für Psychische Gesundheit (DZPG), Bochum, Deutschland

**Keywords:** Psychotherapie, Technologie, Outcomes, Wirksamkeit, Psychiatrische Versorgung, Psychotherapy, Technology, Outcomes, Efficacy, Mental health services

## Abstract

Digitale Interventionen (DI) gewinnen zunehmend als Erfolg versprechende Instrumente zur Unterstützung von Menschen mit Psychosen an Relevanz und ergänzen traditionelle therapeutische Ansätze. In dieser Übersicht werden der Nutzen und die Wirksamkeit aktueller DI (einschließlich Virtual-Reality‑, Smartphone- und Onlineinterventionen) untersucht, die die Behandlungserfolge bei Psychosen verbessern wollen. Während zunehmend Belege auf ihre Wirksamkeit bei der Reduktion positiver, kognitiver und affektiver Symptome im Vergleich zur Standardbehandlung und anderen Therapien hinweisen, liegen nur wenig Erkenntnisse aus großen, kontrollierten Studien vor. Weitere Forschung ist erforderlich, um Interventionsstrategien zu optimieren, langfristige Ergebnisse zu verbessern und Umsetzungsrahmen zu entwickeln, die die Zugänglichkeit im Rahmen der Routineversorgung sicherstellen. Ihre vollständige Integration in die psychiatrische Versorgung erfordert nachhaltige Anstrengungen in Forschung, Politik und klinischer Praxis. Zu den wichtigsten Herausforderungen gehören die Entwicklung autonomer Interventionen, die Erschließung neuer, für Patienten relevanter Behandlungsziele und die Schaffung von Lösungen für eine nachhaltige Finanzierung dieser Anwendungen über klinische Forschungsstudien hinaus.

**Zusatzmaterial online:**

Die Online-Version dieses Beitrags (10.1007/s00115-025-01883-x) enthält eine Übersicht über Begriffsdefinitionen, die verwendeten Suchbegriffe sowie den Algorithmus zur Selektion.

## Abstract (en)

Digital interventions (DI) are emerging as promising tools to support individuals with psychoses and complement traditional therapeutic approaches. This overview examines the usability and efficacy of recently developed DI (including virtual reality, smartphone and online interventions) designed to enhance treatment outcomes in psychoses. While emerging evidence suggests their efficacy in reducing positive, cognitive and affective symptoms in comparison to treatment as usual and other active therapies, evidence from large, controlled trials is still scarce. Further research is needed to refine intervention strategies, enhance long-term outcomes and develop implementation frameworks that ensure accessibility within routine care. Their full integration into mental health services requires sustained efforts in research, policy and clinical practice. Key remaining challenges include the design of autonomic interventions, discovery of new treatment targets relevant to patients and creating solutions for sustainable funding of these applications beyond clinical research studies.

Der Begriff Psychose beschreibt ein weites Symptomspektrum, welches Störungen der Wahrnehmung, des Denkens, der Emotionen und des Verhaltens beinhaltet. Es ist in erster Linie durch Wahnvorstellungen, Halluzinationen und Denkstörungen gekennzeichnet, oft einhergehend mit desorganisiertem Verhalten und Negativsymptomen. Neben der medikamentösen Behandlung spielt auch Psychotherapie eine zentrale Rolle, wobei die kognitive Verhaltenstherapie für Psychosen (KVTp) den am besten belegten Ansatz darstellt [1]. Doch obwohl die derzeitige psychiatrische Versorgung diverse Behandlungsmöglichkeiten für Menschen mit psychotischen Symptomen bietet, ist der Zugang zu spezialisierten Therapien nach wie vor begrenzt und die Anpassung bestehender Angebote an die Bedürfnisse dieser Zielgruppe gestaltet sich schwierig [2, 3].

## Digitale Interventionen für Psychosen

Digitale Technologien liefern neue Möglichkeiten, um psychologische Interventionen auf ansprechende und maßgeschneiderte Weise zu verbessern und neue therapeutische Kontexte zu schaffen, in denen psychologische Kernprozesse in Echtzeit und mit unmittelbarem Feedback angesprochen werden können. Diese Technologien haben das Potenzial, Hindernisse für den Zugang zu psychologischen Therapien zu beseitigen, indem sie jungen Menschen innovative und flexible Plattformen bieten, auf denen sie Erfahrungen austauschen und Unterstützung finden können. Außerdem eröffnen sie neue Wege für die Zusammenarbeit mit Behandlern und Forschern, ermöglichen eine genauere Beurteilung der Person und fördern die Umsetzung von Therapiezielen im Alltag.

Aktuell werden primär drei Formen digitaler Interventionen (DI) für die Behandlung von Menschen mit Psychosen evaluiert:

- telemedizinische und internetbasierte (eHealth) Interventionen,
- App-basierte mobile Gesundheitsanwendungen (mHealth) und
- Virtual-Reality(VR)-Therapien [4].

Virtual Reality hilft dabei, die Kluft zwischen Therapie und alltäglichen Situationen zu schließen, indem sie die Nachbildung realer Umgebungen mit einem hohen Maß an Präzision ermöglicht und somit Patienten befähigt, Therapieerfolge in einem alltagsnahen, aber sicheren Umfeld und mit Unterstützung des Therapeuten zu festigen [5]. mHealth findet in der Regel in Form von Smartphone-Apps Anwendung, wird aber auch für das passive Monitoring behavioraler und physiologischer Parameter verwendet [6]. Smartphones können auch verwendet werden, um Daten wie den Standort, digitale Kommunikationsmuster und andere Variablen zu ermitteln, die mit Selbsteinschätzungsinstrumenten weniger reliabel erfasst werden können.

Digitale Interventionen können entweder als Ergänzung zur bestehenden Behandlung eingesetzt werden oder als eigenständige Interventionen fungieren. Der Deutsche Bundestag verabschiedete 2019 das Gesetz zur digitalen Gesundheitsversorgung (Digital-Versorgungs-Gesetz, DVG; [7]), welches digitale Gesundheitsanwendungen, die bestimmte Kriterien erfüllen, in den Leistungskatalog der gesetzlichen Krankenkassen aufnimmt. Gleichzeitig fördert das Gesetz den Einsatz von Telemedizin sowie die Nutzbarkeit von Gesundheitsdaten für Forschungszwecke.

Zum aktuellen Zeitpunkt existieren keine nach DVG zugelassenen DiGAs für die Behandlung psychotischer Erkrankungen (Bundesinstitut für Arzneimittel und Medizinprodukte, BfArM). Dieses Review bietet daher eine Übersicht über die zentralsten internationalen Entwicklungen im Bereich digitaler Interventionen für Psychosen. Grundlage hierfür bieten die wichtigsten Publikationen der vergangenen 10 Jahre, die den Einsatz digitaler Technologien (z. B. virtuelle Realität, Smartphone-Technologie und Onlineinterventionen) zur Verbesserung der psychologischen Behandlung von Psychosen überprüfen. Darüber hinaus diskutieren wir aktuelle Entwicklungen in Deutschland und sprechen Empfehlungen für die zukünftige Forschung und Implementierung von DI für Psychosen aus.

## Methoden

Es wurde eine systematische Suche in PubMed durchgeführt, um aktuelle DI für Psychosen im Publikationszeitraum zwischen Januar 2014 und Dezember 2024 zu identifizieren (Suchbegriffe siehe Supplementary Material 2). Eingeschlossen wurden alle Interventionen, die Internet, Smartphone oder VR-Technologie verwenden und auf psychoserelevante Mechanismen in klinischen Stichproben abzielen. Ausgeschlossen wurden Studien zur reinen Symptomüberwachung, familienzentrierte Interventionen, Reviews oder Artikel, die nicht in englischer oder deutscher Sprache verfügbar waren.

Die Suche ergab 639 Artikel, durch die 52 Interventionen identifiziert wurden (Selektion siehe Supplementary Material 3). Eine wichtige Limitation ist das noch sehr frühe Forschungsstadium. Beim Großteil der identifizierten Studien handelt es sich um Pilotstudien; nur wenige Interventionen wurden mittels groß angelegter randomisiert-kontrollierter Studien (RCTs) oder Replikationen überprüft.

Das vorliegende Review konzentriert sich daher auf die Interventionen mit den empirisch fundiertesten Ergebnissen, die somit das größte Potenzial für eine Aufnahme in die Gesundheitsversorgung haben.

## Ergebnisse

### Auditive verbale Halluzinationen

#### Blended Intervention

Der Großteil der Evidenz für den Einsatz von VR zur Behandlung von Positivsymptomen konzentriert sich auf Verfolgungswahn und akustische verbale Halluzinationen (AVH; [8]). Einer der am besten etablierten Ansätze für AVH ist die AVATAR-Therapie (AT; Avatar Therapy Ltd, London, UK). Gemäß der Erfindung von Julian Leff nutzt die AT nichtimmersive VR-Technologie, um einen Dialog zwischen dem Patienten und einer digitalen Repräsentation der belastenden Stimme (dem Avatar) zu ermöglichen. Die Intervention besteht traditionell aus 6 Sitzungen, in denen der Patient mittels Unterstützung des Therapeuten die Kontrolle über die Stimme zurückzugewinnen lernt. Der Avatar passt sich dabei nach und nach dem Fortschritt des Patienten an und erkennt dessen Stärken an.

In einer RCT, in der die AVATAR-Therapie mit supportiven Gesprächen verglichen wurde, zeigte sich eine deutliche Verringerung der stimmbezogenen Belastung, des Schweregrads sowie der Stimmenfrequenz in der Therapiegruppe [9]. In einer aktuellen multizentrischen RCT wurde die traditionelle AVATAR-Therapie mit 6 Sitzungen mit einer erweiterten 12-Sitzungs-Adaption und „treatment as usual“ (TAU) allein verglichen. Schweregrad und Belastung durch die Stimme zeigten nach 16 Wochen in beiden Therapiegruppen signifikante Verbesserungen im Vergleich zum TAU, die Stimmfrequenz reduzierte sich nur in der erweiterten Therapiegruppe signifikant [10]. Eine Studie, in der immersive VR zur Erprobung von AT im stationären Rahmen modifiziert wurde, zeigte ebenfalls ermutigende Ergebnisse [11].

Aktuelle Forschungsbemühungen versuchen, die Ergebnisse der ersten AT-RCT von Craig et al. in anderen Versorgungssystemen zu replizieren [12]. In Deutschland läuft derzeit eine klinische Studie zur AT, die zusätzlich zur Therapie ein immersives VR-Element ergänzt, um Behandlungserfolge in soziale Interaktionen zu übertragen, und so stimmbezogene Ängste sowie Vermeidungsverhalten reduzieren soll [13].

#### App-basierte Anwendungen

Die robustesten Belege für den Einsatz von mHealth bei Positivsymptomen, insbesondere AVH, stammen von zwei App-basierten Anwendungen. SAVVy (Swinburne University of Technology, Australien) ist eine mHealth-Intervention, die 4 KVT-basierte Therapiesitzungen mit einer Smartphone-App verbindet, welche dem Nutzer individualisierte Bewältigungsstrategien für den Umgang mit akuten belastenden Stimmen schickt. Die App sammelt Echtzeitdaten zu Stimmen, Auslösern und Bewältigungsversuchen und schlägt darauf basierend funktionale Copingstrategien vor. In den Therapiesitzungen werden die Fortschritte überprüft und mögliche Aktualisierungen durch Patienten und Therapeut in der App vorgenommen. Eine RCT, in dem SAVVy mit TAU verglichen wurde, berichtet positives Nutzerfeedback und hohe Engagementraten, es ergaben sich jedoch keine signifikanten Unterschiede in der primären Outcomevariable des Schweregrads der Stimme [14].

Die Temstem-App (Vrije Universiteit Amsterdam, Niederlande) ist eine autonome mHealth-Intervention für belastende Stimmen und umfasst zwei Sprachspiele: *Silencing*-Spiele vermitteln Strategien zur Zurückweisung von Stimmen, während *Challenging*-Spiele durch Dual Tasking deren Einfluss verringern. Eine unkontrollierte Studie zeigte signifikante Verbesserungen der stimmenbezogenen Belastung [15], in einer RCT ergaben sich jedoch keine signifikanten Gruppenunterschiede im Vergleich zu reiner Symptombeobachtung [16].

### Wahn

#### VR-Therapie

Die VR-basierte Behandlung von Verfolgungswahn folgt dem Angstmodell und nutzt Exposition, um paranoide Überzeugungen zu hinterfragen und zu korrigieren.

GameChange (University of Oxford, UK) ist die erste automatisierte VR-Therapie und zielt darauf ab, agoraphobisches Vermeidungsverhalten und Ängste bei Patienten mit Psychosen zu behandeln. Sie besteht aus 6 Sitzungen, in denen der Patient eine Reihe von Verhaltensexperimenten durchläuft, die seine Befürchtungen in Bezug auf andere Personen infrage stellen. Ein virtueller Therapeut führt den Patienten durch die Übungen und fordert ihn auf, Sicherheitsverhalten abzulegen. Eine multizentrische RCT ergab eine signifikante Verringerung der Vermeidung von und der Belastung in Alltagssituationen in der Therapiegruppe im Vergleich zur Kontrollgruppe [17].

In ähnlicher Weise zielt auch VR Exposure Therapy (VRET; Vrije Universiteit Amsterdam, Niederlande), eine niederländische VR-basierte Intervention mit 16 Sitzungen, darauf ab, paranoide Gedanken zu reduzieren und die soziale Partizipation zu erhöhen. Der Therapeut kann dabei relevante Faktoren wie das Aussehen und die Reaktionen der virtuellen Avatare an die individuellen Befürchtungen anpassen. Während der Sitzungen führt der Therapeut den Patienten durch die Übungen und hilft dabei, paranoide Gedanken, Sicherheitsverhalten und Schadenserwartungen zu hinterfragen. In einer multizentrischen RCT zeigte sich die Intervention bei der Reduktion paranoider Gedanken im Vergleich zum TAU überlegen [18]. Die Ergebnisse einer dänischen multizentrischen RCT, die eine modifizierte 10-Sitzungs-Variante von VRET mit KVTp vergleicht, stehen noch aus [19].

#### Blended-Intervention

SloMo (University of Oxford, UK) ist eine Blended-Intervention, die eine Onlineplattform mit einer Smartphone-App und 8 KVT-Sitzungen kombiniert. Die Intervention zielt darauf ab, paranoide Symptome zu verringern, indem sie vorschnelle Denkstile und deren Auswirkungen auf paranoide Gedanken herausarbeitet und Strategien zum Verlangsamen des eigenen Denkens und zum Umgang mit belastenden Gedanken vermittelt. In einer großen RCT erzielte SloMo nach 12 Wochen eine signifikante Verringerung der paranoiden Gedanken im Vergleich zum TAU, welche jedoch nicht zur Nachuntersuchung nach 24 Wochen gehalten werden konnte [20]. Insgesamt entsprach die Verbesserung der paranoiden Gedanken durch SloMo den Therapieerfolgen traditioneller KVTp, erzielte diese aber in weniger Sitzungen.

#### App-basierte Anwendungen

Die Actissist-App (University of Manchester, UK), ein KVT-basiertes Tool für frühe Psychosephasen, erfasst belastende Erfahrungen und liefert Bewältigungsstrategien in Echtzeit. Die App fokussiert auf Kernmechanismen früher Psychosen wie Halluzinationen, Paranoia, Sozialverhalten und Cannabiskonsum. Während eine Pilotstudie positive Effekte auf Negativsymptome und Stimmung zeigte [21], ergab eine RCT bei Ersterkrankten (FEP) keine signifikanten Verbesserungen gegenüber reiner Symptombeobachtung [22].

##### Metakognitives Training

Basierend auf dem etablierten metakognitiven Training (MKT), welches kognitive Verzerrungen adressiert, die im Zusammenhang mit der Entwicklung von Wahnvorstellungen stehen [23], wurde die Cogito-App (Universität Hamburg, Deutschland) entwickelt. Die frei verfügbare App will den Behandlungserfolg von MKT verbessern. Die App beinhaltet KVT- und MKT-basierte Übungen und fordert Nutzer dazu auf, eine bis zwei Übungen pro Tag durchzuführen. Patienten und Behandler können außerdem personalisierte Übungen hinzufügen. Aktuell wurden noch keine Forschungsergebnisse zur Wirksamkeit von Cogito zur Behandlung psychotischer Symptome veröffentlicht, jedoch belegen Studien aus anderen klinischen Gruppen, dass die App die Reduktion depressiver Symptome sowie die Steigerung des Selbstwertgefühls unterstützt [23].

Eine frühere Studie zu einer App mit ähnlichen Modulen (EviBaS [Universität Hamburg, Deutschland]) zeigte, dass die KVT- und MKT-basierten Übungen zu einer signifikanten Verringerung der Positivsymptomatik im Vergleich zu einer Wartelistenkontrollgruppe führten [24]. Die Ergebnisse einer MKT-basierten Onlineselbsthilfeintervention zur Verringerung der Frequenz von und der Belastung durch AVH befinden sich aktuell im Preprint. Während die Studie auf den Primärvariablen keine Unterschiede zwischen Therapie- und Wartelistengruppe feststellte, zeigte sich die Häufigkeit von Halluzinationen insgesamt in der Therapiegruppe signifikant reduziert [25].

### Soziale Fertigkeiten und soziale Leistung

Gemäß geltenden S3-Leitlinien ist die Förderung sozialer Kompetenzen ein wichtiger Bestandteil der Behandlung von Psychosen, insbesondere in frühen Stadien. Klassisches soziales Kognitions- und Interaktionstrainings (SCIT) in Form von Gruppentherapie leidet jedoch häufig an Schwierigkeiten bei der Rekrutierung und der Therapieteilnahme, was vermutlich auf motivationale Defizite oder soziale Ängste zurückzuführen ist. Die VR-Technologie könnte diesen Problemen entgegenwirken. Daher modifizierten bereits mehrere Forschungsteams das traditionelle SCIT als VR-basierte Intervention (VR-SCIT; Zhejiang University, China), bei der gamifizierte Aufgaben zur Vermittlung von Psychoedukation und zum Training verschiedener Bereiche der sozialen Kompetenz eingesetzt werden.

Shen et al. [26] verglichen ihr automatisiertes VR-SCIT mit sowohl traditioneller SCIT als auch mit einer Wartelistenkontrolle. Während die Behandlungsgruppen im Vergleich zur Kontrollgruppe ähnlich signifikante Verbesserungen der sozialen Funktionsfähigkeit erzielten, zeigte sich VR-SCIT bei der Verbesserung von Emotionswahrnehmung und Metakognitionen überlegen und führte zu einer höheren Therapieteilnahme als das traditionelle SCIT. Nijman et al. entwickelten Interactive Social Cognition Training in VR (DiSCoVR; VRelax BV, Groningen, Niederlande), ein von Therapeuten geleitetes SCIT, das immersive VR für Trainingsmodule verwendet. Eine multizentrische RCT, das DiSCoVR mit VR-basierter Entspannung verglich, fand keine signifikanten Unterschiede in Bezug auf soziale Kognition [27].

Horyzons (Orygen, Parkville, Australien) ist eine innovative webbasierte Therapie, die darauf abzielt, Rezidive bei Personen nach ihrer ersten psychotischen Episode zu verhindern und die Genesung aufrechtzuerhalten. Die Plattform besteht aus interaktiven, KVT-basierten psychoedukativen Elementen und einer moderierten sozialen Plattform. Basis hierfür ist der Befund, dass das Rückfallrisiko und der Übergang in chronische Verläufe über den Behandlungszeitraum spezialisierter FEP-Dienste hinaus bestehen. Eine australische RCT evaluierte die Wirksamkeit von Horyzons als Nachsorge nach FEP-Intervention. Nach einem 18-monatigen Interventionszeitraum zeigten Personen in der Horyzons-Gruppe signifikant verbesserte Beschäftigungs- oder Bildungserfolge sowie niedrigere Hospitalisierungsraten im Vergleich zu Personen der Kontrollgruppe [28]. Es gab jedoch keine Gruppenunterschiede im primären Outcome der sozialen Funktionsfähigkeit.

### Negative Symptome

V‑NEST (King’s College London, UK) ist eine innovative Blended-Intervention, die spezifisch auf Anhedonie und Motivationsdefizite im Zusammenhang mit Negativsymptomen abzielt [29]. Die 12 Therapiesitzungen integrieren VR-gestützte individuell zugeschnittene Motivationsaufgaben, die der Patient mit Unterstützung des Therapeuten bewältigen soll. Erste Ergebnisse zeigten ein hohes Maß an Akzeptanz und Engagement, selbst bei Teilnehmern mit schweren und stark einschränkenden Negativsymptomen. Außerdem ergaben sich Behandlungseffekte in der Erreichung persönlicher Therapieziele (großer Effekt) und in der Reduktion der Negativsymptomatik insgesamt (kleiner Effekt).

## Diskussion

Trotz der Fortschritte in der Psychotherapieforschung und Technologie sind bestehende digitale Interventionen für Psychosen noch weit von der Integration in die Routineversorgung entfernt. Die aktuellen Anforderungen der DiGA-Verordnung in Deutschland bestimmen, dass zulässige digitale Interventionen vollständig autonom anwendbar und ihre Wirksamkeit durch robuste empirische Evidenz belegt sein müssen. Die meisten DI für Psychosen sind nach wie vor Blended-Interventionen, die die digitale Applikation als ergänzendes Instrument von Psychotherapiesitzungen einsetzen. Demnach sind viele DI im Rahmen der DiGA-Verordnung ungeeignet. Auch die hier genutzte Einteilung von Interventionen in Symptomcluster spiegelt eine weitere Schwierigkeit wider. Die meisten DI für Psychosen adressieren einen spezifischen Symptomkomplex, nicht aber das gesamte Störungsbild. Dementsprechend kann gemäß DVG keine Behandlungsempfehlung für das gesamte diagnostische Spektrum ausgesprochen werden. Dabei ist fraglich, ob komplexe Störungsbilder wie Schizophreniespektrumsstörungen je umfassend durch einzelne DI behandelt werden können.

Die Integration von DI in die psychiatrische Versorgung hängt maßgeblich von ihrer Kompatibilität mit bestehenden Systemen ab. Während das Einrichten des Equipments Zeit und Fachwissen erfordert, wird die Technologie immer benutzerfreundlicher und mobiler [8]. Eine wirksame Umsetzung erfordert die Schulung von Behandlern, Ressourcen sowie Aufklärung der Patienten über evidenzbasierte Behandlungen. Dabei bietet die Integration von DI in die Routineversorgung wichtige Chancen, z. B. in der Überbrückung von Wartezeiten, in der Nachsorge von Patienten mit leichter oder mittlerer Symptomschwere und in der Prävention neuer Episoden bei (teil)remittierten Krankheitsbildern. Eine solche Erweiterung des Behandlungsspektrums ist im Hinblick auf die Barrieren, mit denen diese Patientengruppe oft konfrontiert wird [2, 3], von großer Bedeutung.

Ein weiteres Hauptproblem ist die begrenzte Verfügbarkeit empirisch validierter DI für Psychosen außerhalb von Forschungsprojekten. In Übereinstimmung mit den Ergebnissen einer aktuellen Metaanalyse [1] weisen die hier diskutierten Interventionen Effektgrößen im Bereich von 0,1 bis 1,49 auf. Obwohl immer mehr digitale Programme entwickelt werden, werden nur wenige Interventionen umfassend evaluiert. Auch die hohen Entwicklungskosten bei nicht tragfähigen Finanzierungsmodellen stellen weiterhin eine große Hürde dar. Um die Wirksamkeit von DI zu maximieren, müssen Forscher im Entwicklungsprozess eng mit betroffenen Personen zusammenarbeiten (d. h. partizipative Forschung). Hierzu gehört auch der Einbezug qualitativer Evaluationen von DI. In einem aktuellen Review qualitativer Studien über digitale Interventionen für Menschen mit Psychosen wurden mehrere zentrale Vorteile identifiziert. Dazu gehörte, dass sie Betroffenen helfen, ihre Erfahrungen besser zu verstehen, den Umgang mit belastenden Symptomen zu verbessern und das Gefühl sozialer Zugehörigkeit zu fördern. Zusammengenommen stärken diese Prozesse das Gefühl von Empowerment, das für den Genesungsprozess von Menschen mit Psychose eine entscheidende Rolle spielt [30].

## Fazit für die Praxis

- Behandler benötigen Schulung und fortlaufende Unterstützung.
- Das Therapiedesign sollte den Nutzerbedürfnissen entsprechen und Engagement fördern.
- Der Einsatz von Digitalen Interventionen (DI) bei Psychose verspricht eine effektivere Nutzung von Wartezeiten bzw. das Potenzial zur Verkürzung von Wartelisten sowie die breitere Verfügbarkeit von kognitiver Verhaltenstherapie für Psychosen (KVTp).
- DI können die Behandlungseffizienz steigern und die Arbeit des Behandlers erleichtern.
- Weitere randomisiert-kontrollierte Studien (RCTs) sind notwendig, um Wirksamkeit und Kosteneffektivität über verschiedene Gruppen und klinische Stadien hinweg zu prüfen.
- Für die evidenzbasierte Umsetzung ist eine strengere Validierung für Sicherheit und Wirksamkeit erforderlich.
- Die Zugänglichkeit sollte für verschiedene Nutzergruppen sichergestellt werden.
- Die Unterstützung auf Systemebene erfordert eine nachhaltige Finanzierung und Infrastruktur.
- Verbleibende Herausforderungen sind eigenständige Anwendungen, neue Behandlungsziele und Betroffenenpartizipation.

## Supplementary Information

ESM1 Infobox Begriffsdefinitionen

ESM2 Suchbegriffe

ESM3 Selektion
