# Supplementary material for: Digital interventions for psychoses: current opportunities and challenges
Source: Nervenarzt. 2025 Aug 11;96(5):439–44. [Article in German] doi: 10.1007/s00115-025-01883-x (PMC12411582; doi:10.1007/s00115-025-01883-x)
Supplement: Supplementary file 2 — ESM2 Suchbegriffe [file 115_2025_1883_MOESM2_ESM.pdf]

Die Suchbegriffe umfassten "virtuelle Realität" ODER "VR" ODER "Smartphone" ODER "mobile App" ODER "internetbasiert" ODER "webbasiert" ODER "eHealth" ODER "mHealth" UND "Psychose", ODER "Schizophrenie" UND "Intervention" ODER "Therapie" ODER "Behandlung". Förderfähige Studien betrafen nutzerzentrierte psychologische
