# Supplementary figures and images for: Digital interventions for psychoses: current opportunities and challenges
Source: Nervenarzt. 2025 Aug 11;96(5):439–44. [Article in German] doi: 10.1007/s00115-025-01883-x (PMC12411582; doi:10.1007/s00115-025-01883-x)

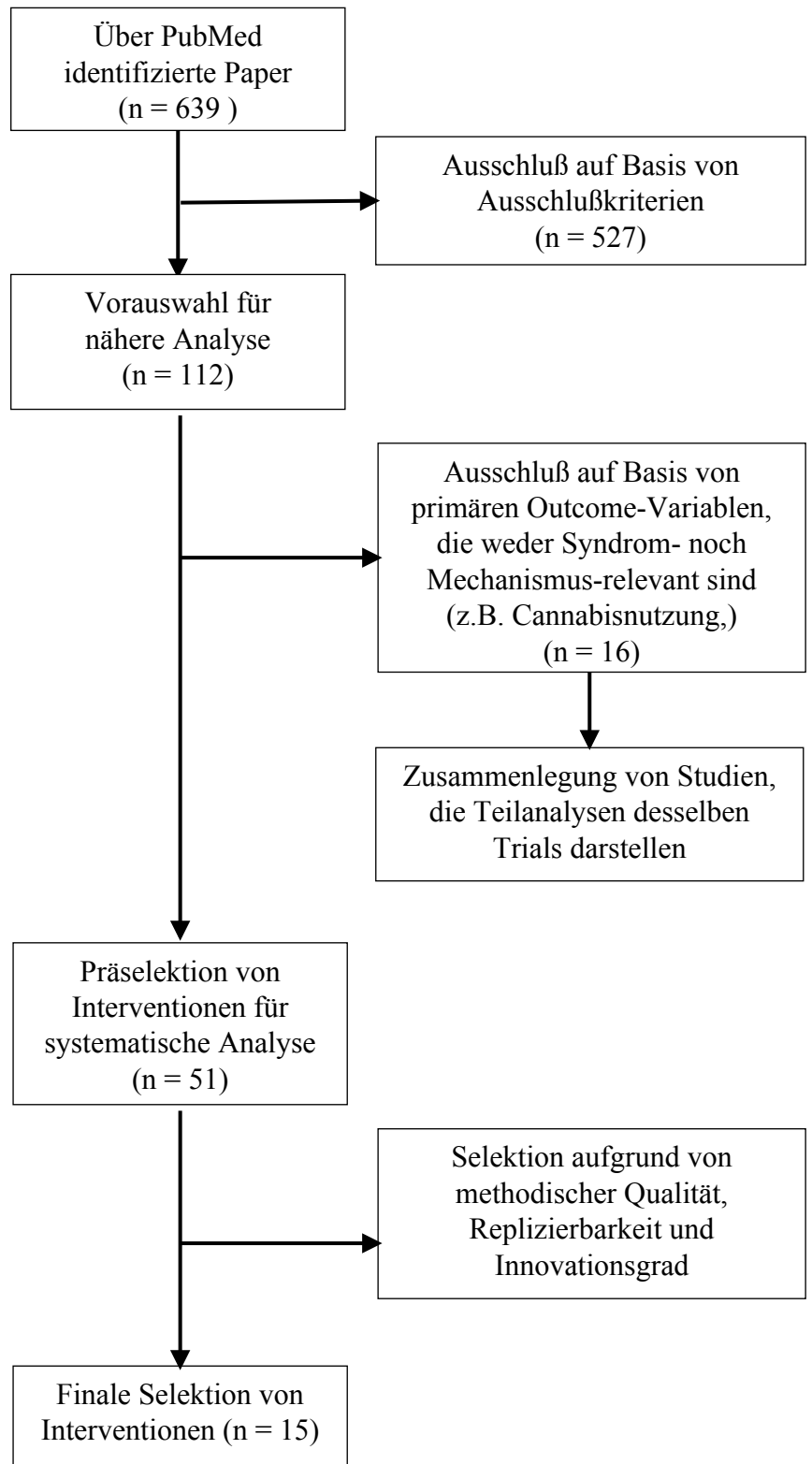

Supplement: Supplementary file 3 — ESM3 Selektion [file 115_2025_1883_MOESM3_ESM.pdf]
